# New Treatment in Advanced Thyroid Cancer

**DOI:** 10.1155/2012/391629

**Published:** 2012-10-22

**Authors:** Dario Giuffrida, Angela Prestifilippo, Alessia Scarfia, Daniela Martino, Stefania Marchisotta

**Affiliations:** Department of Medical Oncology, Mediterranean Institut of Oncology, Via Penninazzo, 7, 95029 Viagrande, Italy

## Abstract

Thyroid cancer is the most common endocrine tumor. Thyroidectomy, radioactive iodine, and TSH suppression represent the standard treatment for differentiated thyroid cancer. Since chemotherapy has been shown to be unsuccessful in case of advanced thyroid carcinomas, the research for new therapies is fundamental. In this paper, we reviewed the recent literature reports (pubmed, medline, EMBASE database, and abstracts published in meeting proceedings) on new treatments in advanced nonmedullary and medullary thyroid carcinomas. Studies of many tyrosine kinase inhibitors as well as antiangiogenic inhibitors suggest that patients with thyroid cancer could have an advantage with new target therapy. We summarized both the results obtained and the toxic effects associated with these treatments reported in clinical trials. Reported data in this paper are encouraging, but further trials are necessary to obtain a more effective result in thyroid carcinoma treatment.

## 1. Introduction

Thyroid cancer is rare, but is the most prevalent endocrine malignancy tumor. In 2002, in the USA 141,000 cases occurred and 35,300 deaths were estimated [1]. Among different parts of the world there is a 10-fold difference in incidence for women, but only a 3-fold difference for men [2]. 

The differences between the sexes declines after the middle age, but still three out of four cases arise in women. The most well-established cause of thyroid cancer is the exposure to ionizing radiations, particularly during childhood. Iodine deficiency influences thyroid function directly as well as indirectly, through a reduction of thyroid hormones levels and a consequent increase in TSH secretion. Chronic iodine deficiency is firmly established as a risk factor for goiter and follicular thyroid cancer, while some aetiological studies suggested that iodine supplementation programmes could increase the incidence of papillary thyroid cancer by inducing iodine excess. Supplementation effects are likely to be confused by diagnostic procedures improvement and therefore there may be not a biological background at the basis of this phenomenon [3]. Thyroid cancer is a heterogeneous disease that is classified into differentiated thyroid carcinoma (DTC), anaplastic thyroid carcinoma (ATC) and medullary thyroid carcinoma (MTC). DTC and ATC together are classified as nonmedullary thyroid cancer (NMTC). DTCs are the most common histotype (85%), and include papillary (70%) and follicular (10%–15%) as well as subtypes like Hurthle cell carcinomas. Although activating point mutations of the TSH receptor have been discovered in 60–70% of benign toxic adenomas, a pathogenetic role for these mutations in malignant transformation has been excluded or rarely reported [4]. In the last two decades, the molecular basis of thyroid cancer have been well characterized and the critical genetic pathways involved in the development of specific tumors histotype have been elucidated. Around 20–25% of thyroid medullary carcinomas can be attributed to genetic factors [5]. In particular, germ-line mutations in the RET gene are responsible for the hereditary tumour syndrome (i.e., multiple endocrine neoplasia type 2, MEN 2) which includes three subgroups, MEN 2A, MEN 2B, and familial medullary thyroid carcinoma (FMTC), depending on the tissue involved. Follicular cell proliferation and function is physiologically regulated by thyroid-stimulating hormone (TSH). Most of the DTC are slowly progressive and frequently cured with adequate surgical management and radioactive iodine (131-I) ablation therapy (RAI), when identified at an early stage. Metastatic DTC that is untreatable by surgery or refractory to radioactive iodine therapy is associated with poor survival. MTC and, especially, ATC metastasize up to the 50% of diagnosticated cases, giving a worst prognosis. ATC is one of the most aggressive neoplasm in humans with a mortality rate over 90% and a mean survival of 6 months after diagnosis [6, 7]. Standard treatments in some cases of advanced differentiated thyroid cancer and medullary thyroid cancer (radiotherapy and/or chemotherapy) have been unsatisfactory and therefore new therapies are necessary. In the past decade, multiple clinical trials have been carried out thanks to an increased knowledge of the biological basis of thyroid cancer and to development of new treatments that target biological substrates. This paper will focus on current clinical trials and recent therapies on specific target involved in thyroid carcinogenesis. 

## 2. Molecular Target Therapy in Advanced Thyroid Cancer

Recent advances in molecular biology resulted in significant improvement in our understanding of the pathogenesis of thyroid carcinoma

Gene rearrangements involving the RET and TRK proto-oncogenes have been demonstrated as causative events specific for a subset of the papillary histotype. Recently, another oncogene, BRAF, has been specifically associated with PTC with a frequency around 40%. Mutated forms of the H-ras, K-ras, and N-ras oncogenes are found in differentiated thyroid cancer, but the same mutation are also described in benign thyroid lesion.

RET-activating point mutations have been found exclusively in medullary thyroid carcinoma (MTC) and these mutations are observed in both sporadic MTC and FMTC.

 All the identified mutation on RAS, RET, TRK, and BRAF genes involve MAP kinase activation. An abnormal activation of this pathway is one of the most studied mechanisms of thyroid tumorigenesis. In a lower percentage, other abnormalities have been reported to be involved in thyroid tumorigenesis such as DNA methylation [8] and gene deletions in chromosomes 11q13 and 3p [9]. 

RAS-activation induces cell division and inhibits cell differentiation. The expression of p21, the RAS-encoded protein, plays an important role in the intracellular signal transduction from the cell surface to the nucleus where it is able to activate genes expression that induces cell proliferation [10]. In thyroid neoplastic cell proliferation RAS role is still poorly known. It has been hypothesized that activated p21 could interact with some thyroid-specific transcription factors such as TTF1 or PAX-8 [11]. RAS activating point mutations have been found in 3 hot spots localized in the codons 12, 13, and 61. RAS oncogene point mutations account for nearly 40% of benign and malignant follicular thyroid tumours while they are rare in the papillary histotype [12, 13]. Interestingly, RAS mutations are more frequent in thyroid tumors of subjects living in countries where iodine intake is inadequate [14]. 

The RET proto-oncogene is located on chromosome 10q11-2. It encodes for a tyrosine kinase transmembrane receptor involved in the activation of the MAP kinase cascade. The proto-oncogene is normally expressed in a variety of neural cell lineages including thyroid C cells and adrenal medulla but it is not expressed, or it is expressed at very low levels, in normal thyroid follicular cells [15]. RET oncogene activation may be generated either by a fusion rearrangement of the tyrosine kinase domain of RET gene and the 5′ domain of other genes [16] or by activating point mutations [17]. RET/PTC rearrangements have been reported only in PTC [18] and in some cases of benign follicular adenomas [19]. Activating RET-point mutations have been exclusively found in MTC [17]. Several RET/PTC rearrangements have been described and all of them are characterized by the fusion of the RET tyrosine kinase domain with a housekeeping gene triggering the constitutive RET expression in the follicular cell [20–22]. RET/PTC rearrangements are related to ionizing radiation exposure which is a well-recognized risk factor for PTC. The evidence of an increasing incidence of RET/PTC rearrangements in childhood post-Chernobyl thyroid carcinomas [23] and the possibility of determining RET/PTC rearrangements *in vitro *in thyroid cells experimentally exposed to ionizing radiation [24] is a clear proof in favour of a causative connection between radiation exposure and these chromosomal alterations. Despite this evidence, RET/PTC rearrangements have also been reported in unirradiated thyroid lesions [25]. The prevalence of RET/PTC rearrangements in thyroid tumors of patients who had no history of neck irradiation ranges from 2.5 to 35% among different series [16, 23, 26–30]. The identification of RET/PTC rearrangements in microPTCs suggests that this is an early event in thyroid carcinogenesis [29]. On the other hand, RET/PTC positive tumors do not show a tendency of progression to poorly or undifferentiated tumor phenotype [31]. Germline RET point mutations in MTC are mainly localized in the tyrosine kinase domain and in the cysteine domain of the gene. Recently several other noncysteine mutations have been described, usually correlated with less aggressive phenotypes [32]. The point mutation determines a constitutive activation of the tyrosine kinase receptor and, as consequence, a continuous stimulus to cell proliferation. In thyroid tumors alteration of RET pathway have been found not only on mutation/overexpression of RET gene, but have been attributed to downstream protein.

**Figure 1:**
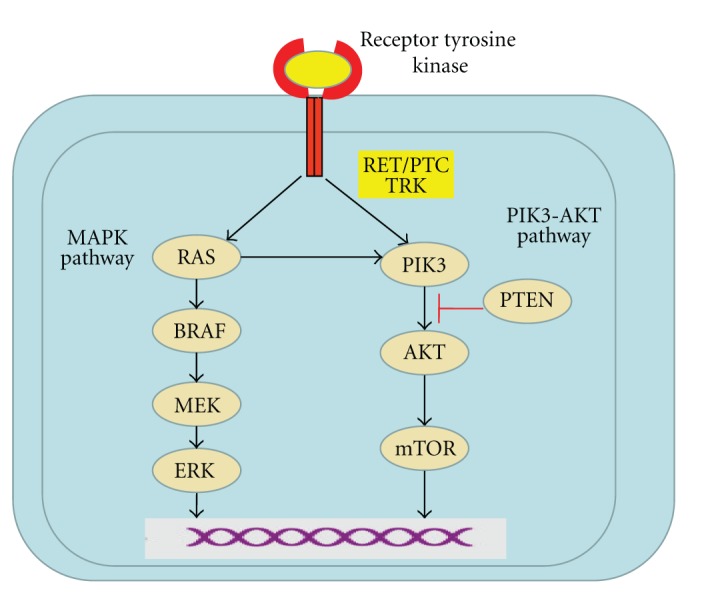
signaling pathways in thyroid cancer.

Recently, an activating mutation of the B isoform of the Raf kinase gene, located on exon 15, which results in a valine to glutamic acid substitution at amino acid 600 (BRAF^V600E^ mutation) has been found to be the most common mutation in PTC (Figure 1). [33] This mutation has a key role in leading to a constitutively activated state of the gene and thus tumorigenesis. Recently, *BRAF* ^V600E^ has emerged as a promising prognostic factor in the risk stratification of PTC and it has showed an association between *BRAF* mutation and high-risk clinical-pathological characteristics of PTCs [34].

## 3. Standard Treatment of Thyroid Cancer

Radioiodine (131-I) therapy has been used in the treatment of patients with well-differentiated tumors (papillary or follicular). Thyroid cancer tissue has a unique ability to uptake iodine from blood. Like iodine, radioiodine is uptaken and concentrated in thyroid follicular cells by specific membrane transporters. Compared with normal thyroid follicular cells, thyroid cancer cells have reduced expression of the transporter, which may account for the low 131-I uptake in thyroid cancer tissue.

131-I causes acute thyroid-cell death by emission of short path-length (1 to 2 mm) beta rays. 131-I uptake by thyroid tissue can be visualized by gamma radiation scanning. 131-I must be uptaken by thyroid tissue to be effective, resulting in an absence of response in patients whose thyroid cancers do not concentrate iodide, for example, patients with medullary cancer, lymphoma, or anaplastic cancer. Indications for 131-I administration after thyroidectomy in patients with differentiated thyroid cancer include ablation of residual normal thyroid tissue, adjuvant therapy of subclinical micrometastatic disease, and treatment of clinically apparent residual or metastatic thyroid cancer. The efficacy of radioiodine for both scanning and treatment depends upon patient preparation, tumor-specific characteristics, sites of disease, and dose [33, 35–37]. 

## 4. New Treatment Modalities in Thyroid Cancer

In a near future, Tyrosine Kinase Inhibitors (TKIs) may open a new era in the radioactive iodine refractory DTC and advanced MTC patients treatment. However, the published clinical trials are relatively limited compared to other malignancies and there is only one reported phase III trial in thyroid cancers and many others phase III are ongoing. The difficulty in enrollment of an adequate number of patients to these clinical trials may be a possible reason for this. It may be possible to overcome this difficulty by multi-institutional trials. On the other hand, there is no proof yet that TKIs improve overall survival. Moreover, having a relatively high number of significant undesirable effects, (see Table 1) patients must be selected carefully before starting the therapy. Randomised clinical trials for several agents are ongoing. 

We examined the results and the adverse events for each TKIs used in thyroid-cancer-targeted therapy, reported in literature. 

### 4.1. Sunitinib (SU1248)

Sunitinib is a multitargeted tyrosine kinase inhibitor (TKI). Targets of the drug include vascular endothelial growth factor receptor (VEGFR) types 1 and 2, platelet-derived growth factor receptors, c-KIT, FLT3, and RET. The inhibitory effect of the drug on VEGF and RET makes it a rational candidate for the therapy of DTC and MTC. Somatic mutations of the proto-oncogene RET are critical in the development of MTC. In addition, elevated serum levels of vascular endothelial growth factor are also associated with poor prognosis in papillary carcinoma of the thyroid. 

Sunitinib is currently approved for the therapy of renal cell carcinoma and gastrointestinal stromal tumor (GIST) on an intermittent treatment schedule. Actually the effect of sunitinib on DTC and MTC patients has been reported only on phase II trials, as phase III trials are absent. 

Preliminary results from an open-label phase II trial in patients with progressive DTC or MTC reported partial response in 13% of 31 DTC patients, and disease stabilization in 68% of DTC and 83% of MTC patients [38]. Treatment consisted of 6-week cycles of sunitinib malate 50 mg everyday on a 4-week on/2-week off schedule. Primary endpoint was clinical response rate evaluated by RECIST and biochemical response rate. 

The most common drug-related adverse events included fatigue (79%), diarrhea (56%), palmar-plantar erythrodysesthesia (53%), neutropenia (49%), and hypertension (42%). Grade 3-4 toxicity included neutropenia (26%), thrombocytopenia (16%), hypertension (16%), fatigue (14%), palmar-plantar erythrodysesthesia (14%), and gastrointestinal tract events (14%) [38]. Additionally, in an open-label phase II trial in patients with progressive DTC or MTC 18 patients were enrolled (3 MTC, 15 DTC) [39]. Treatment consisted of sunitinb 37.5 mg daily until tumor progression or prohibitive toxicity. The primary endpoint was response rate per RECIST criteria. Secondary endpoints included FDG-PET scan response rate (defined as 20% reduction from baseline SUV) after 7 days of treatment, toxicity, overall survival, duration of response, and time-to-tumor progression. Preliminary results showed that 44% of patients had FDG-PET response. All these patients had DTC. Grade 3 toxicities included neutropenia (28%), leukopenia (17%), anemia (6%), thrombocytopenia (6%), fatigue (11%), hand-foot syndrome (11%), pain (11%), gastrointestinal bleeding (11%), diarrhea (6%), mucositis (6%), and atrial fibrillation (6%) of the patients. There have been no grade 4 toxicities. 

Recently, in a phase II study, sunitinib was administered at a dose of 37.5 mg/day in continuous schedule [40]. Thirty-five patients were evaluated with sunitinib; twenty-four patients underwent evaluation by FDG-PET both at baseline and after 7 days of sunitinib therapy. 

Eight of 29 patients with DTC and 3 of 6 patients with MTC achieved a RECIST response (response rate, 28% and 50% for DTC and MTC, resp.). There were 1 complete response (3%) and 10 partial responses (28%). In addition, 16 patients (46%) had stable disease. 

The median time to progression (TTP) was 12.8 months, and the decline in the uptake of fluorodeoxyglucose (FDG) at 7 days of treatment with sunitinib was superior in those patients who subsequently achieved positive radiological response (by RECIST criteria). 

The most common toxicities seen included fatigue (11%), neutropenia (34%), hand/foot syndrome (17%), diarrhea (17%), and leukopenia (31%). One patient on anticoagulation died of gastrointestinal bleeding. 

Tumors were highly metabolically active by FDG-PET, with median lesion SUV of 7.9, indicating an aggressive phenotype. In fact the presence of FDG-avid tumors is strongly predictive of a more aggressive course of the disease and associated with a 5-year OS of less than 50% [41].

Carr et al. [40] attempted to correlate the results of a FDG-PET scan one week after therapy initiation with a subsequent response to therapy, based on data showing that a decline in FDG uptake could be an early indicator of response in other diseases treated by sunitinib. 

It was observed that there is a significant association between average SUV percent change and RECIST response. Patients with partial/complete response and stable disease had a significant decline in average SUVs compared with patients with progressive disease. This could provide a very useful method to predict treatment benefit, particularly when using an expensive therapy in a clinical situation where stable radiologic disease is of unclear significance. It is possible, and perhaps likely, that an FDG-PET done later than 1 week from treatment initiation would have been a better predictor of benefit and may merit further investigation. 

Another open question is about type of schedule of Sunitinib. In fact, it was administered at a dose of 37.5 mg/day in a continuous schedule, while in renal cell carcinoma and gastrointestinal stromal tumor (GIST) Sunitinib is currently approved on an intermittent treatment schedule. 

Therefore phase III clinical trials are necessary to define their accurate clinical benefit and the best schedule of treatment. 

### 4.2. Sorafenib (Bay 43-9006)

Sorafenib (BAY 43-9006) is an oral, small-molecule TKI targeting VEGF receptors 2 and 3, RET (including most mutant forms that have been examined), and BRAF. In preclinical studies, sorafenib prevented the growth of the TPC1- and TT-cell lines, which contain the RET/PTC1 and C634W RET mutations, respectively. 

The effect of sorafenib on DTC and MTC patients has been reported on 4 nonrandomized phase II studies which used a dose of sorafenib 800 mg/day as a single agent in patients with DTC refractory to radioactive iodine. At the moment no phase III trials have been reported. 

In the 30 patients treated by the group of Gupta-Abramson et al. a median PFS of 18.4 months was achieved: 7 (23%) patients achieving an objective radiological partial response and 16 patients (53%) achieving disease stabilization of more than 6 months [42]. 

In a more recent study, similar results were observed in 41 patients with PTC. In these patients, the objective radiological response rate was 15%, and disease stabilization was observed in 56% of patients [43]. The median PFS was 15 months. 

In another study, a total of 34 patients with thyroid cancer were treated (15 MTC, 19 DTC) with an objective response rate of 25% in DTC and 18% in MTC at 12 months [44]. In a more recent study conducted in 32 DTC patients, the partial response rate achieved was 25%, and a stabilization of disease was observed in 34% of patients at 26 weeks [45]. Most adverse effects occurring in these 4 studies were consistent with the already-known safety profile of the drug; the majority of toxicities found were grade I and II and easily manageable with a delay or dose reduction of sorafenib administration. Taken together, these results formed the scientific basis for the launch of a phase III registration termed DECISION (Study of Sorafenib in Metastatic or Locally Advanced, Refractory Patients with Thyroid Cancer RAI). The study compared the administration of sorafenib versus placebo in 380 patients with radioiodine-refractory DTC with PFS as the primary endpoint (NCT00984282). This study has just completed recruitment, and results are awaited with interest. 

The anti-RET activity of sorafenib makes MTC a potential therapeutic target for this drug as well. 

Preliminary results have been reported from open-label phase II study in patients with metastatic MTC [46]. Although partial response was observed in only 6% of patients with sporadic MTC, stable disease lasting more than 6 months was reported in 62%. A high frequency of side effects was noted, including flushing, diarrhea, weight loss, alopecia, hand-foot syndrome, and rash. Severe adverse events included a pulmonary embolus, hypokalemia, hypertension, hyponatremia, joint pain, and thrombocytopenia. 

Anticipating synergy between sorafenib's ability to inhibit MAPK signaling and the RAS-blocking effects of the farnesyltransferase inhibitor tipifarnib, a phase I trial was performed of the combination of these drugs. The maximum tolerated doses of sorafenib and tipifarnib were 200 and 100 mg twice daily, respectively. In the 22 patients with DTC treated, median PFS was 20 months [47].

### 4.3. Vandetanib (ZD6474)

Vandetanib is a small-molecule tyrosine kinase inhibitor of vascular endothelial growth factor receptor-2 (VEGFR-2), epidermal growth factor receptor (EGFR), and rearranged-during-transfection (RET-) dependent signaling. 

MTC is a rare disease for which vandetanib was granted orphan-drug designation and for which there were previously no approved therapies. In the majority of cases of MTC, there is activation of the RET proto-oncogene, and both VEGFR and EGFR signaling pathways may also contribute to the pathogenesis. 

On the basis of the preclinical demonstration that vandetanib inhibited most RET-point mutations, a multicenter, open-label phase II trial studied the efficacy of the drug in patients with metastatic familial forms of MTC. Thirty patients were enrolled, starting therapy with vandetanib, 300 mg daily. Confirmed partial response was reported in 21% of these patients, the median duration of response at data cutoff was 10.2 months. Calcitonin levels dropped by more than 50% in most patients (80%), but blocking RET may lead to a direct inhibition of calcitonin-gene expression, independent of tumor volume changes [48]. Adverse events were predominantly grade 1 or 2, and the most common events included diarrhea, fatigue, rash, and nausea. The most common grade 3 adverse events were QT prolongation and diarrhea, nausea, and hypertension. There were grade 4 adverse events of azotemia or muscle weakness, which were not considered by the investigator to be related to vandetanib. All of these events were managed with dose interruptions or reductions. 

To assess the potential efficacy of a lower dose of vandetanib, Robinson and colleagues conducted a second single-arm phase II study in a similar population of patients with hereditary MTC to evaluate the activity of a 100 mg dose of vandetanib [49]. This study comprised 19 patients and demonstrated that the lower dose of vandetanib also has activity in this patient population. The objective tumor response rate was 16%, with a median duration of response of 6 months. The median PFS could not be determined because of an insufficient number of progression events. However, only 16% of the patients had a reduction in calcitonin levels of at least 50% from baseline. 

Vandetanib 100 mg/d was well tolerated in the majority of patients in this study, most adverse events were of Common Terminology Criteria for Adverse Events (CTCAE) grade 1 or 2 and were manageable. Diarrhea, fatigue, and rash were the most common adverse events reported. 

On the basis of the results of the phase II studies in hereditary MTC, Wells and colleagues initiated a randomized, placebo-controlled phase III study (ZETA) of vandetanib in patients with MTC. The ZETA study enrolled patients with both hereditary and sporadic MTC. A total of 331 patients were randomized to receive vandetanib 300 mg or placebo in a 2 : 1 ratio [50]. 

The ZETA study demonstrated a clinically significant benefit for vandetanib in prolonging PFS, with a statistically significant hazard ratio (HR) = 0.46 (95% confidence interval = 0.31–0.69; *P* = 0.0001). This HR represents a 54% reduction in the risk of progression for patients randomized to vandetanib. The median PFS for patients randomized to placebo was 19 months, whereas the median PFS for patients randomized to vandetanib was not reached but was estimated to be approximately 30 months. In addition to the benefits with respect to PFS, vandetanib also induced objective tumor responses in 45% of patients. Among the patients randomized to placebo, 13% (13 patients) had an objective tumor response according to the intention-treat analysis, but 12 of these 13 responses occurred only after the patients had switched over to open-label vandetanib. Significant decreases in calcitonin and CEA levels were seen in patients randomized to vandetanib, with 69% of patients on the vandetanib arm experiencing a calcitonin response (decline of at least 50% from baseline) and 52% having a CEA response, as compared to 3% and 2%, respectively, in those on placebo. 

Almost all the patients randomized to vandetanib on the ZETA study experienced at least one adverse event, and 55% experienced an event of Common Terminology Criteria for Adverse Events (CTCAE) grade 3 or higher. The most commonly reported side effects included rash (particularly photosensitivity), diarrhea, fatigue, and nausea, whereas the most severe toxicities included asymptomatic QT interval prolongation, rash, and diarrhea. The most common side effect of vandetanib in the study was diarrhea, which could have been difficult to distinguish from disease-related diarrhea in some cases. 

In conclusion, vandetanib has clinical antitumor activity in patients with advanced or metastatic hereditary MTC and in April 2011, the US Food and Drug Administration (FDA) approved it for the treatment of symptomatic or progressive medullary thyroid cancer (MTC) in patients with unresectable locally advanced or metastatic disease.

### 4.4. Motesanib (AMG 706)

Motesanib is an oral inhibitor of multiple kinases,including VEGFR-1, 2, and 3 as well as the wild and mutant forms of the membrane receptor RET. In a phase I trial a 50% overall response rate was observed in patients with advanced thyroid carcinoma. Based on these results, a multicenter phase II trial was initiated, testing the efficacy of motesanib therapy in patients with progressive or symptomatic MTC. In this study the, median progression free survival was 40 weeks. Of 91 patients with progressive or symptomatic MTC who initiated therapy, only 2% had a confirmed partial response, but another 48% had stable disease for at least 24 weeks. The most common adverse events found at any grade were diarrhea (41%), hypertension (27%), fatigue (41%), and weight loss (22%) [51]. 

### 4.5. XL281

XL281 is a small molecule with potential antineoplastic activity specifically inhibits RAF kinases, located downstream from RAS in the RAS/RAF/MEK/ERK kinase signaling pathway, which may result in reduced proliferation of tumor cells. RAS mutations may result in constitutive activation of the RAS/RAF/MEK/ERK kinase signaling pathway, and have been found to occur frequently in human tumors. Preliminary data with the oral administration of this compound described prolonged a stable disease in 5 patients with PTC; of the 2 patients whose tumor were substained to contain BRAF mutations, both remained stable after more than 1 year of therapy [52]. 

### 4.6. Axitinib (AG013736)

Axitinib (AG-013736) is an oral inhibitor that effectively blocks VEGF receptors at subnanomolar concentrations, but notably not the RET kinase. 

One of five patients with thyroid carcinoma included in a phase I trial experienced tumor shrinkage, which however, was not qualified as a PR [53]. A phase II trial by Cohen et al. [54] studied the efficacy of axitinib in advanced or metastatic thyroid carcinoma of any histology (*n* = 60). A PR was seen in 30% of the patients. Stable disease lasting more than 16 weeks was reported in 38%. Objective responses were noted in all histological subtypes with a PR rate of 31% in patients with DTC and 18% in patients with MTC. Median PFS was 18.1 months. Common adverse events included diarrhea, hypertension, fatigue, decreased appetite, nausea, dysphonia, hand-foot syndrome, weight decreased, vomiting, and asthenia. 

Exploratory analyses of soluble biomarkers showed increases in serum VEGF levels, a recognized phenomenon of effective angiogenesis inhibition. Given the absence of inhibitory activity against RET or other mutated kinases that are oncogenic in thyroid carcinoma, the efficacy of axitinib suggests that VEGFR-mediated angiogenesis is likely the primary mechanism by which the other anti-VEGFR inhibitory agents function. Currently ongoing is a multicenter, open-label phase II study to determine the efficacy of axitinib in patients with metastatic DTC refractory to doxorubicin, or if doxorubicin therapy is contraindicated. 

### 4.7. XL184

XL184 is a small molecule designed to inhibit multiple tyrosine kinases receptors, specifically MET and VEGFR2. MET is a tyrosine kinase receptor that plays a key role in cellular proliferation, migration, and invasion as well as angiogenesis These biological processes contribute to the transformation, progression, survival, and metastasis of cancer cells. The MET pathway is frequently activated in tumors through MET amplification, mutation, and overexpression, as well as through overexpression of its ligand HGF. Expression of VEGF has been observed in a variety of cancers and has been associated with the stimulation and growth of new blood vessels to support the tumor. MET and VEGFR2 are important driving forces in angiogenesis, implicated in the ability of tumors to overcome hypoxia following angiogenesis inhibition. A phase I study was conducted in patients with metastatic solid malignant tumors including 37 MTC. The endpoint of the study included a dose escalation, the analysis of XL184 pharmacokinetics, safety, and RECIST response. Ten patients with MTC achieved partial response. Additionally 41% of MTC patients had stable disease for at least 6 months. Patients responsiveness was independent to the RET mutation status, an indication that the drug is active in patients without RET-activating mutations. A phase III trial, comparing XL184 with placebo, is ongoing [55]. 

### 4.8. Pazopanib (GW 786034)

Pazopanib is a potent and selective multitargeted receptor tyrosine kinase inhibitor of VEGFR-1, VEGFR-2, VEGFR-3, PDGFR-a/*β*, and c-Kit that blocks tumor growth and inhibits angiogenesis. It has been approved for renal cell carcinoma by the U.S. Food and Drug Administration. Pazopanib may also be active in ovarian cancer and soft tissue sarcoma. Pazopanib also appears effective in the treatment of non-small-cell lung carcinoma and thyroid cancer. In a phase II study, pazopanib administered at a dose of 800 mg/day induced a radiographic response rate of 49% in 37 patients with DTC who had disease progression over the previous 12 months. Progression-free survival was 11.8 months. The most frequent toxiticies found were fatigue (78%), skin rash (75%), diarrhea (73%), and nausea (73%) [56]. 

### 4.9. Lenvatinib (E7080)

Lenvatinib is an oral tyrosine kinase inhibitor targeting VEGFR1-3, FGFR1-4, RET, KIT, and PDGFR*β* [57, 58]. 

It is a synthetic, orally available inhibitor of vascular endothelial growth factor receptor 2 (VEGFR2, also known as KDR/FLK-1) tyrosine kinase with potential antineoplastic activity. Lenvatinib blocks VEGFR2-activation by VEGF, resulting in inhibition of the VEGF-receptor-signal-transduction pathway, decreased vascular endothelial cell migration and proliferation, and vascular endothelial cell apoptosis; thus inhibits both VEGFR2 and VEGFR3 kinases. 

In a phase II trial, 58 patients with refractory DTC were treated [59] with a starting dose of Lenvatinib 24 mg once daily in 28-day cycles untill disease progression. Primary end-point was response rate (RR) by RECIST. 

Patients receiving prior VEGFR-directed treatment (*n* = 17) had an RR of 41%; while patients with not prior VEGFR-directed treatment (*n* = 41) had an RR of 54%. Median PFS was 12.6 months. 

However, dose reduction was required in 35% of patients, and 23% of them discontinued treatment due to toxicity. The most frequent grade 3 or 4 toxicities that led to dose reductions were hypertension (10%), proteinuria (10%), decreased weight (7%), diarrhea (10%), and fatigue (7%). 

This results formed the scientific basis for the launch of a phase III trial in which DTC refractory to radioactive iodine were randomized to receive lenvatinib or placebo. 

Moreover, recently, therapeutic strategies have been investigated to study the ability of the proteasome inhibitor bortezomib to inhibit growth in ATC cell lines. Bortezomib was used as a single agent or in combination with TNF-related apoptosis-induced ligand to obtain the destruction of chemoresistant neoplastic thyrocytes and may represent a promising therapeutic agent in the treatment of ATC [60]. 

## 5. Discussion

Standard treatment for differentiated thyroid cancer is based on total thyroidectomy, radioactive iodine, and TSH suppression. Despite the generally good prognosis of differentiated thyroid carcinoma, about 20% of patients will develop metastatic disease which fails to respond to radioactive iodine, exhibiting a more aggressive behavior. 

Systemic chemotherapies for advanced or metastatic nonmedullary and medullary thyroid carcinomas have been of only limited effectiveness. For patient with differentiated or medullary carcinomas unresponsive to conventional treatments, novel therapies are needed to improve disease outcomes. 

Aberrations in RET/PTC-RAS-RAF-MAPK pathway are present in a high percentage of thyroid cancer, as well as angiogenesis switch alterations and involvement of other receptor tyrosine kinases, such as VEGFR or c-Met. Because of the oncogenic roles of activated BRAF, RET, and RET/PTC kinases, the hypothesis that specific targeting of these kinases could block tumor growth was suggested. Targeted agents against the VEGF receptor and the MAP kinase pathway are amongst the most promising thus far (see Table 2) [61]. 

Although most small-molecule VEGF receptor antagonists also inhibit RET, the efficacy of axitinib and pazopanib to induce objective responses in the absence of any significant anti-RET activity suggests that RET may not be as important a target for therapy as VEGFR. Unfortunately, eventual progression despite antiangiogenic VEGFR blockade suggests emergence of alternate pathways to promote tumor growth and metastasis. 

The aim of the introduction of these targeted therapies is to extend life duration while assuring a good quality of life. Toxicities of many of these new therapies, although less life-threatening than cytotoxic chemotherapies, are common and can be dose limiting, and clinicians should be familiar with recognizing and managing the side effects if they intend to use these agents. 

While significant progress has been made in understanding some of the mechanisms underlying tumorigenesis and in translating that knowledge into various treatment modalities, numerous challenges remain in testing targeted therapies against refractory thyroid cancer. 

Selecting a primary endpoint for phase II and III trials is difficult. Although the Response Evaluation Criteria in Solid Tumors (RECIST) is a methodology for standardizing the reporting of therapeutic response categories in cancer patients target therapies often produce a cytostatic, rather than cytotoxic response, in which case tumor shrinkage may not be seen, even in cases of highly effective therapy. 

This has led many Phase II trials to revert to progression-free survival (PFS), rather than response rate (RR), as the primary imaging metric of efficacy. However, determining progression times and rates, rather than response rates, requires longer monitoring periods (especially in cases of effective therapies). Actually, no novel treatment has been demonstrated to advance the time of survival for patients with thyroid cancer. 

Thus, objective responses using RECIST or PFS as an endpoint in phase II trials or overall survival as an endpoint in a phase III trial may not be optimal. 

Likewise, many of the studies are measuring serum levels of thyroglobulin, calcitonin, or CEA to determine if these biomarkers may be used as an additional tool to evaluate response to therapy. As seen in the studies previously described, however, these markers are only partially usefull and may not be a reliable indicator of disease responsiveness. Further studies are needed, to understand the relationship between targeted molecular therapies and their direct effects on the synthesis or secretion of tumor-marker proteins.

Moreover another challenge is selecting appropriate patients for phase II and III clinical trials. An argument can be made to restrict eligibility of patients into clinical trials to those with PD in the 6 or 12 months prior to study entry so that attribution of SD as an objective response to targeted therapy may be interpretable. Furthermore, patients with an overall indolent cancer may be spared the toxicities of targeted therapies. A significant limitation of this approach, however, is that patients diagnosed at an advanced stage with severe or symptomatic tumor burden who desperately need therapy may not be eligible for the trials due to inability to prove PD at the study entry. 

Additionally new studies should point out the possibility to use politherapy than monotherapy and cytotoxic chemotherapies in combination with target therapy to obtain more response that has not completely been reached in any of the actual trials. 

However, the published clinical trials are relatively sparse compared to other malignancies and there is only one published phase III trial yet in thyroid cancers. A possible reason is the difficulty in accrual of enough number of patients to these clinical trials. 

It may be possible to overcome this difficulty by multi-institutional trials recruiting patients from several centers and working in multidisciplinary team (medical oncologist, endocrinologist, specialist in nuclear medicine, radiologist, surgeon, phatologist, molecular biologist, etc.) to enlarge the number of patients in clinical studies, to optimize the aim of protocols, to improve the characterization of tumor tissues, and to improve the tolerance of treatment.

## Figures and Tables

**Table 1:** Most frequent (all grade) adverse events of tyrosine kinase inhibitors used in thyroid cancer.

Adverse event	Sunitinib [37, 39]	Sorafenib [41–45]	Vandetanib [47–49]	Motesanib [50]	Axitinib [53]	Pazopanib [55]	Lenvatinib [58]
Hypertension	22%	48%	33%	27%	28%	—	64%
Diarrhea	37%	77%	57%	41%	48%	73%	45%
Fatigue	45%	48%	43%	41%	50%	78%	55%
Weight loss	—	54%	30%	22%	25%	64%	43%
Nausea	—	22%	37%	26%	33%	73%	44%
Hand-foot skin reaction	35%	91%	—	—	15%	—	—
Rash	—	73%	46%	—	15%	75%	—

**Table 2:** Summary of results of the most important clinical trials conducted in advanced thyroid carcinoma.

Drug	Target	Type of study (ref)	Histology	No. of patients	PR (%)	SD (%)
Sunitinib	VEGFR 1-2 PDGF, RET,	Phase II [37]	DTC	31	13%	68%
	c-KIT, FLT3	Phase II [39]	DTC (29), MTC (6)	35	31%	46%

		Phase II [41]	DTC	30	23%	68%
		Phase II [42]	DTC	41	15%	56%
Sorafenib	VEGFR 1-2 PDGF, RET RAF MAPK	Phase II [43]	MTC (15)/DTC (19)	34	15%	74%
		Phase II [44]	DTC	32	25%	34%
		Phase II [45]	MTC	15	6%	62%

		Phase II [47]	MTC	30	21%	53%
Vandetanib	VEGFR 1-2 EGFR, RET	Phase II [48]	MTC	19	16%	53%
		Phase III [49]	MTC	231	44%	20%

Motesanib	VEGFR 1-2-3 EGFR, RET	Phase II [50]	MTC	91	2%	48%

Axitinib	VEGF	Phase II [53]	MTC (11)/DTC (45)Other (4)	60	30%	38%

XL 184	VEGF, MET, RET, c-KIT, FLT3	Phase I [54]	MTC	37	29%	41%

Pazopanib	VEGFR-1, VEGFR-2, VEGFR-3, PDGFR-c-Kit	Phase II [55]	DTC	37	49%	

Lenvatinib	VEGFR1-3, FGFR1-4, RET, KIT PDGFR*β*	Phase II [58]	DTC	58	50%	
